# Novel anti-thrombotic agent for modulation of protein disulfide isomerase family member ERp57 for prophylactic therapy

**DOI:** 10.1038/srep10353

**Published:** 2015-06-03

**Authors:** Guozhen Cui, Luchen Shan, Lin Guo, Ivan Keung Chu, Guohui Li, Quan Quan, Yun Zhao, Cheong Meng Chong, Zaijun Zhang, Pei Yu, Maggie Pui Man Hoi, Yewei Sun, Yuqiang Wang, Simon MingYuen Lee

**Affiliations:** 1 State Key Laboratory of Quality Research in Chinese Medicine and Institute of Chinese Medical Sciences, University of Macau, Macao, China; 2 Department of Bioengineering, Zhuhai Campus of Zunyi Medical University, Guangdong, Zhuhai, China; 3 Institute of New Drug Research, Collage of Pharmacy, Jinan University, Guangzhou, China; 4 Key Laboratory of Cardiovascular Medicine Research, Ministry of Education, Harbin Medical University, Harbin, China; 5 Department of Chemistry, The University of Hong Kong, Hong Kong, China

## Abstract

Protein disulfide isomerase (PDI) family members including PDI and ERp57 emerge as novel targets for anti-thrombotic treatments, but chemical agents with selectivity remain to be explored. We previously reported a novel derivative of danshensu (DSS), known as ADTM, displayed strong cardioprotective effects against oxidative stress-induced cellular injury *in vitro* and acute myocardial infarct *in vivo*. Herein, using chemical proteomics approach, we identified ERp57 as a major target of ADTM. ADTM displayed potent inhibitory effects on the redox activity of ERp57, inhibited the adenosine diphosphate (ADP)-induced expressions of P-selectin and αIIbβ3 integrin, and disrupted the interaction between ERp57 and αIIbβ3. In addition, ADTM inhibited both arachidonic acid (AA)-induced and ADP-induced platelet aggregation *in vitro*. Furthermore, ADTM significantly inhibited rat platelet aggregation and thrombus formation *in vivo*. Taken together, ADTM represents a promising candidate for anti-thrombotic therapy targeting ERp57.

Cardiovascular diseases (CVDs) remain the leading causes of morbidity and mortality globally. The underlying causes of CVDs are complex and multi-factorial involving many risk factors such as abnormal blood lipid, high blood cholesterol levels, overweight and sedentary lifestyle^1^,^2^. In particular, platelet dysfunction and aberrant thrombogenesis (thrombus formation) are final underlying pathophysiological processes in the development and progression in CVDs including atherosclerosis, thrombotic complications, stroke and myocardial infarction^3^. Current anti-platelet agents such as aspirin and clopidogrel are effective at decreasing platelet activation and subsequent risk of atherothrombotic disease, but these agents irreversibly inhibit platelet activation and lead to risks of unwanted hemorrhages^4^,^5^. Furthermore, the multi-factorial nature of CVDs urges us to look for versatile pharmacological agents with fewer side effects.

We have reported previously that a novel cardioprotective compound known as ADTM was synthesized in our laboratory by conjugating two well-known active ingredients, DSS and tetramethylpyrazine (TMP), which are present in Chinese medicinal plants, danshen (*Salvia miltiorrhizae*) and chuanxiong (*Ligustium wallichii Franch*)^6^. ADTM displayed strong cardioprotective effects against oxidative stress-induced cellular injury and acute ischemic myocardial infarct in rat, partly through activation of the anti-oxidative stress Nrf2/heme oxygenase-1 (HO-1) pathway^7^. Several registered pharmaceutical products containing danshen and/or chuanxiong such as “Danshen Dripping Pill” and “Guanxinning Injection” have been widely used in China to treat CVDs. The “Danshen Dripping Pill” was approved by the US Food and Drug Administration (FDA) to undergo Phase III clinical trials in the treatment of CVDs^8^. Clinical studies indicated that these pharmaceutical products are effective in treating angina pectoris, a symptom of ischemic heart disease^9^. It is believed that active ingredients in danshen and chuanxiong induce beneficial therapeutic effects by the promotion of better blood circulation^10^,^11^. Therefore, we postulated that ADTM might also display anti-platelet and anti-thrombotic properties.

Several members of the protein disulfide isomerases have been shown as important players in the regulation of platelet functions and thrombus formation including PDI, ERp72, ERp57 and ERp5^12^,^13^,^14^. However, which member in the family represents the best anti-thrombotic drug target is largely unknown^13^. The prototypical member PDI is a multifunctional redox chaperone protein that plays critical roles in a variety of diseases including platelet aggregation and thrombus formation *in vivo* and *in vitro*^15^,^16^,^17^. More recently, it is shown that blood platelets also secreted ERp57 (also known as GRP58 and PDIA3) in response to vascular injury to trigger platelet activation and aggregation and emerges as a novel target for anti-platelet aggregation and anti-thrombosis^18^,^19^,^20^. Human ERp57 has 32% amino acid sequence homology with PDI, and exhibits 40% identity with the a-b-b´-a´domains of ERp72^21^. However, selective chemical agents for ERp57 are not yet available.

In this study, we further identified the drug target of ADTM as ERp57 by using a chemical proteomics approach analyzing liquid chromatography linked to tandem mass spectrometry (LC-MS/MS) profile of pull-down proteins bound to a biotinylated-ADTM analogue (BAA). The anti-platelet aggregation and anti-thrombosis activities of ADTM were determined both *in vitro* and *in vivo*. The results provided promising evidences for the potential of developing novel anti-CVD and anti-thrombotic agents with selectivity at the protein disulfide isomerases by using ADTM.

## Results

### Protein disulfide isomerases are identified as targets of ADTM by chemical proteomics

**Figure 1:**
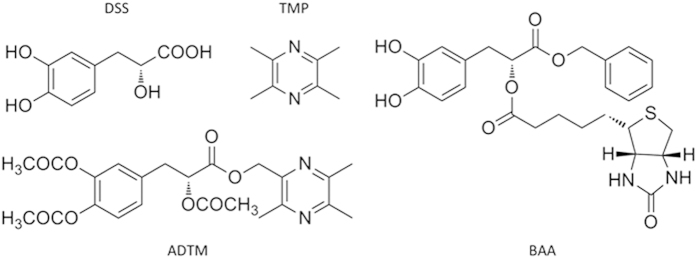
Chemical structures of DSS, TMP, ADTM, and BAA.

**Figure 2:**
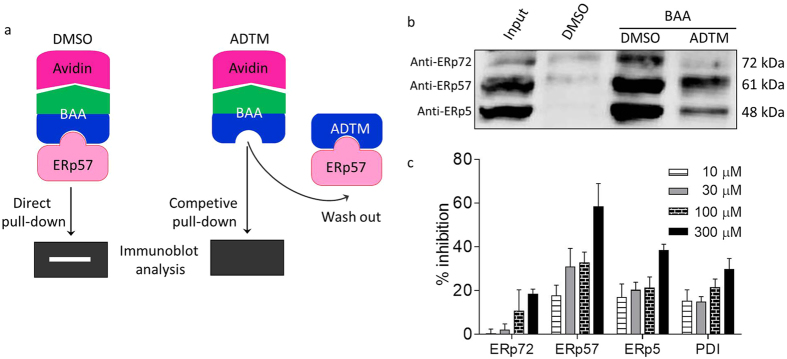
Target validation and the inhibition of PDI members by ADTM. (**a**) Schematics of the target validation. (**b**) Proteins isolated from rat platelets were pretreated with DMSO or excess amount of untagged ADTM for 2 h, followed by treatment with BAA for another 2 h, and compound-protein-complexes were enriched with NeutrAvidin-agarose resin. The precipitates were detected by immunoblot for indicated proteins of PDI family. (**c**) ADTM selectively inhibits ERp57. The recombinant PDI, ERp57 and ERp72 proteins were incubated with different concentrations of ADTM for 100 min, and its catalytic activity was determined in insulin reduction assay.

In order to isolate the protein targets that bound to ADTM, a biotin-conjugated ADTM analogue (BAA, Fig. 1) was designed and synthesized in our laboratory as described in Supplementary Methods (Supplemental Fig. 3). BAA (300 μM) was incubated with rat blood platelet lysates and the BAA-protein complexes were pulled down with NeutrAvidin-agarose followed by protein profiling using LC-MS/MS. It was observed that BAA could bind to various proteins involved in platelet function, including glycoprotein 5 (a potential marker of thrombotic platelet activation^22^,^23^), superoxide dismutase and xanthine oxidase (regulators of vascular redox signaling^24^). In particular, four platelet aggregation-associated proteins were identified with >95% protein identification probability (Supplementary Table S1), including ERp72, ERp57 ERp5 and PDI, which are glycoprotein-specific members of the PDI family related to platelet function and redox homeostasis^25^,^26^. We confirmed the identity of the pulled-down proteins as ERp72, ERp57 and ERp5 with their respective antibodies by using immunoblot analysis (Fig. 2b). Furthermore, the specificity of the binding between BAA and these targets (ERp72, ERp57 and ERp5) could be demonstrated by the competition with untagged ADTM in excess (Fig. 2a). The molecular docking analysis demonstrated that the free binding energy to the active cavity of ERp57 (PDB: 3F8U) is −6.5 kcal/mol. ADTM may form hydrogen bonds with side chains of ARG47, ILE453 and PHE450, respectively, in the catalytic center (CYS406/CYS409) of ERp57 (Supplemental Fig. 2). ERp72, ERp57, ERp5 and PDI are identified as important new targets in redox homeostasis of blood platelet aggregation^12^, therefore we further evaluated the effects of ADTM on the reducing activities of the PDI family members. As shown in Fig. 2c, ADTM exhibited potent inhibition on the redox activity of ERp57 in a concentration-dependent manner (IC_50_ = 100 to 300 μM). ADTM inhibited the activities of ERp72, ERp5 and PDI to a much lesser extent (18%, 38% and 30% of inhibition at 300 μM, respectively), displaying differential inhibitory activity at ERp57 (order of potency as follows: ERp57 > ERp5 > PDI > ERp72). ERp57 inhibition by ADTM was irreversible after dilution of the ERp57-ADTM complex (Supplemental Figure 1), indicating that ADTM may covalently bind ERp57.

### ADTM inhibits platelet aggregation *in vitro*

**Figure 3:**
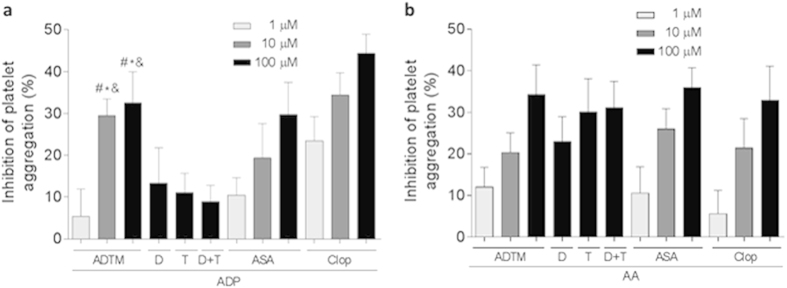
Inhibitory effects of ADTM on ADP-and AA-induced platelet aggregation *in vitro*. Platelets were pretreated with or without the indicated concentrations of the tested compounds, ADTM (1–100 μM), DSS (100 μM), TMP (100 μM), aspirin (1–100 μM) and clopidogrel (1–100 μM) for 5 min at 37 °C and the percentage (%) of platelet aggregation inhibition normalized to DMSO treated samples (vehicle control) was determined as described in Materials and Methods. The platelets were stimulated with ADP (**a**), and AA (**b**). Data are expressed as mean ± SD, ^*^P < 0.01 compared with the 100 μM DSS group. ^#^*P* < 0.01 compared with the 100 μM TMP group, ^&^*P* < 0.01 compared with the 100 μM DSS + TMP group, n ≥ 3/group. D = DSS, T = TMP, ASA = aspirin, clop = clopidogrel.

It was observed that ADTM exhibited concentration-dependent inhibition on ADP-induced platelet aggregation (Fig. 3a) as well as AA-induced platelet aggregation (Fig. 3b) *in vitro* and its effects were comparable to first-line clinical anti-platelet drugs, aspirin and clopidogrel. The parent compounds DSS and TMP, alone or in combination, could only inhibit AA-induced platelet aggregation but not ADP-induced platelet aggregation. These results demonstrated that ADTM exhibited broad-spectrum anti-platelet activities.

### ADTM inhibits the expression of P-selectin

**Figure 4:**
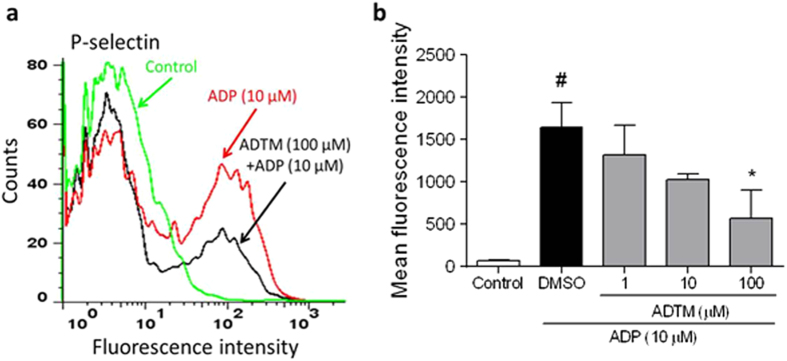
ADTM inhibited the expression of P-selectin. Whole blood was treated with DMSO (0.1%) as a control or the indicated concentrations of ADTM (1–100 μM) for 5 min at 37 °C. After that the platelets were activated using ADP (10 μM). Flow cytometry histogram (**a**) and the quantification of the data (**b**) show that ADTM inhibits P-selectin binding to ADP-activated platelets. ^#^*P* < 0.01 compared with the control group, ^*^P < 0.01 compared with the ADP group. Data are representative of three independent experiments.

To further investigate the underlying mechanism of ADTM anti-platelet activity, we examined the effect of ADTM on the expression of P-selectin (also known as CD62P), which is an adhesion molecule expressed on the surface of platelet and is a critical marker of platelet activation, by using immunoreactivity and flow cytometry analysis. We observed that ADTM concentration-dependently inhibited ADP-induced expression of P-selectin and attenuated the expression of P-selectin by more than 65% at 100 μM (Fig. 4a and 4b).

### ADTM inhibits activation of αIIbβ3 and disrupted ERp57/αIIbβ3 interaction

**Figure 5:**
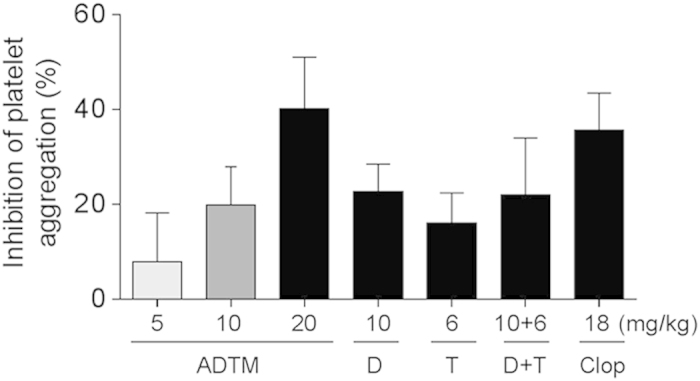
ADTM inhibits activation of αIIbβ3 integrin and disrupts the ERp57/αIIbβ3 complex on platelets. Representative flow cytometry histogram (**a**) and the quantification of the data (**b**) show that ADTM inhibits PAC-1 binding to ADP-activated platelets. (**c**) ADTM disrupts the ERp57/αIIbβ3 complex. Whole blood was treated with DMSO (0.1%) as a control or 100 μM ADTM for 5 min at 37 °C. After that the platelets were activated using ADP (10 μM). Platelet lysates were prepared for immunoprecipitation (IP) and probed with anti-ERp57 antibody for immunoblot analysis (IB). The precipitates were also blotted with anti-αIIbβ3 antibody to ensure that equal amounts of αIIbβ3 were pulled down under each condition. ^#^*P* < 0.01 compared with the control group, ^*^P < 0.01 compared with the ADP group. Data are representative of three independent experiments.

Recently, ERp57 has been reported to directly interact with αIIbβ3 integrin and regulate its function in platelet aggregation^20^. In line with this, we observed that ADTM could modulate αIIbβ3 and concentration-dependently inhibited ADP-induced αIIbβ3 activation, by using flow cytometry analysis with PAC-1, a monoclonal antibody that binds only the activated form of αIIbβ3 (Fig. 5a,b). We further examined the action of ADTM on the ERp57/αIIbβ3 interaction by immunoprecipitation with anti-αIIbβ3 antibody, followed by detection of ERp57 immunoreactivity using immunoblotting. It was found that ADTM disrupted the interaction of ERp57 with αIIbβ3, both in the presence and absence of ADP (Fig. 5c).

### ADTM increases vasodilator-stimulated phosphoprotein (VASP) phosphorylation and HO-1 protein level

**Figure 6:**
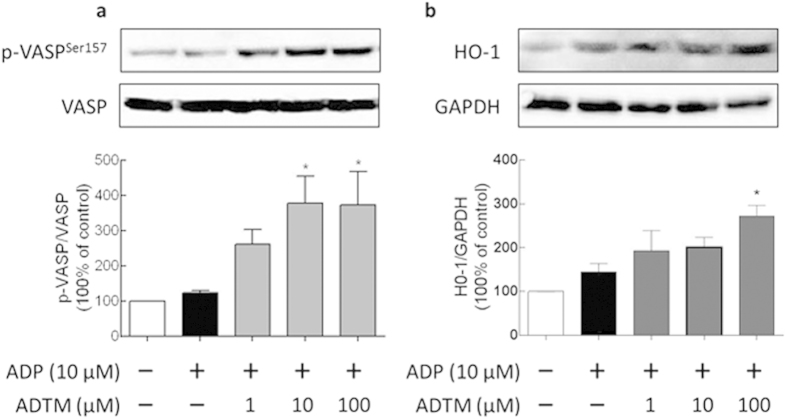
Effects of ADTM on VASP phosphorylation and HO-1 protein Level. Rat platelets (1 × 10^8^ platelets/ml) were incubated with DMSO and various concentrations of ADTM for 10 min followed by treatment with ADP for 5 min. Proteins were extracted from the rat platelets and then analyzed by immunoblot using antibodies against VASP and HO-1 as described in Materials and Methods. ADTM increased VASP phosphorylation (**a**) and HO-1 protein expression (**b**). ^*^P < 0.05 compared with the ADP-stimulated group.

In platelets, the phosphorylation of VASP is involved in the down-regulation of platelet aggregation accompanied by inhibition of platelet integrin αIIbβ3 activation^27^,^28^. As shown in Fig. 6a, pretreatment with ADTM (10 and 100 μM) stimulated approximately three-fold increase in the phosphorylation of VASP. In addition, we also observed that ADTM increased the protein expression of HO-1, an anti-oxidative stress microsomal enzyme which when up-regulated could inhibit platelet-dependent thrombus formation (Fig. 6b)^29^.

### Inhibition of platelet aggregation and thrombus formation by ADTM *in vivo*

**Figure 7:**
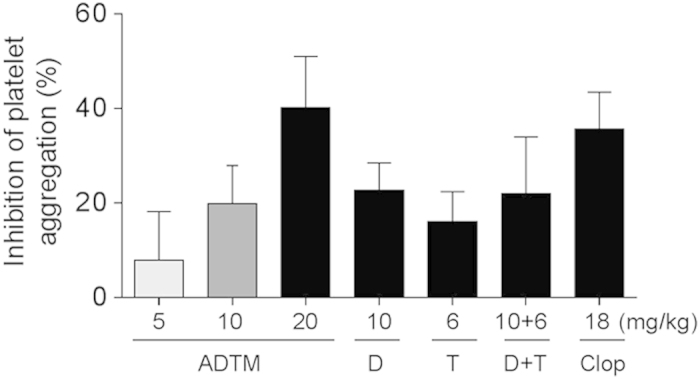
ADTM inhibits platelet aggregation-induced by ADP in a concentration-dependent manner *in vivo*. In order to compare the activities of ADTM, DSS, TMP, DSS + TMP and clopidogrel at a similar blood concentration, 20 mg/kg of ADTM (MW: 444), 10 mg/kg of DSS (MW: 220) and 6 mg/kg of TMP (MW: 136) alone and in combination, and 18 mg/kg of clopidogrel (MW: 322) were administered by IV injection once daily. This resulted in approximately the same blood concentration of ADTM, DSS, TMP, DSS + TMP and clopidogrel in rats *in vivo*. Five days after drug treatment, blood was withdrawn and ADP-induced platelet aggregation was measured. Data are expressed as mean ± SD, n ≥ 6/group. D = DSS, T = TMP, clop = clopidogrel, MW = molecular weight.

**Figure 8:**
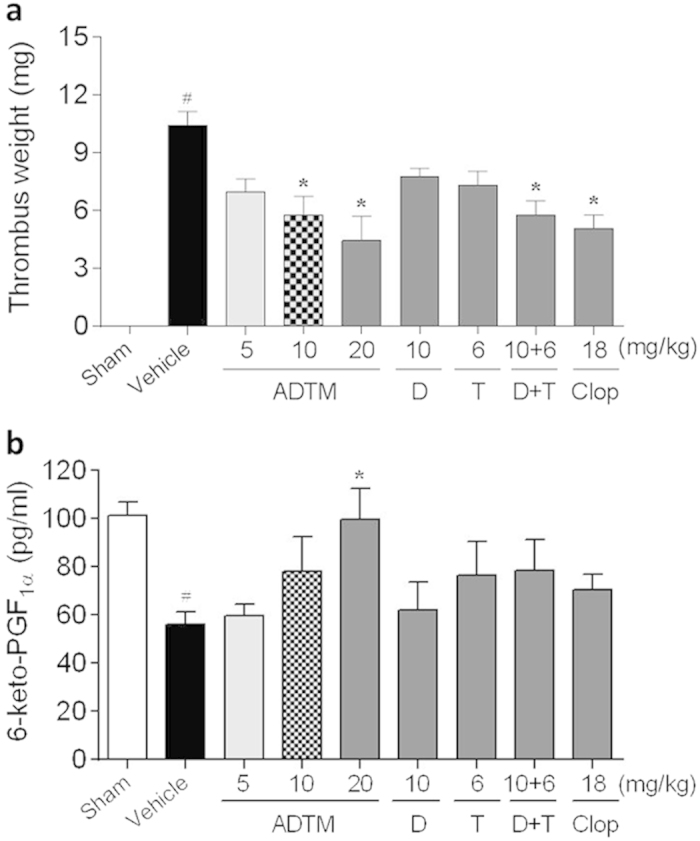
Anti-thrombotic effect of ADTM in the FeCl_3_-induced rat thrombosis model. The anti-thrombotic effect of ADTM in the FeCl_3_-induced inferior vena cava thrombosis model (**a**). In order to compare the activities of ADTM, DSS, TMP and clopidogrel at similar blood concentrations, the compounds were administered by IV injection as described in Fig. 7. The effect of ADTM on plasma 6-Keto-PGF_1a_ concentration in the FeCl_3_-induced thrombosis model (**b**). Data are expressed as mean ± SD, ^#^*P* < 0.01 compared with the sham group, ^*^P < 0.05 compared with the vehicle group, n = 5–7/group, D = DSS, T = TMP, clop = clopidogrel.

Various concentrations of ADTM were administered in rats *in vivo* (10 –20 mg/kg, 5 days, intravenous, IV) and the platelet aggregation activity and the thrombus formation were evaluated. As shown in Fig. 7, ADP-induced platelet aggregation was significantly compromised (>40% reduction) in rats treated with ADTM (20 mg/kg). Similarly, ADTM also exhibited significant anti-thrombotic effect *in vivo* as shown in the ferric chloride (FeCl_3_)-induced venous thrombosis (Fig. 8a). The inhibitory effects of ADTM in platelet aggregation and thrombus formation were comparable to clopidogrel. Furthermore, FeCl_3_ induced significant decrease in plasma 6-Keto-PGF_1α_ (an indicator of plasma PGI_2_, an intrinsic inhibitor of platelet aggregation) and the treatment of ADTM (20 mg/kg, 5 days, IV) abolished the reduction of FeCl_3_-induced 6-Keto-PGF_1α_ in a concentration-dependent manner (Fig. 8b), giving more evidence of the anti-thrombotic properties of ADTM.

## Discussion

Anti-thrombotic therapy plays an essential role in the course of treatment of various cardiovascular disorders (CVDs) because platelet dysfunction and thrombogenesis are common underlying factors in the development and progression in CVDs, particularly atherosclerosis, myocardial infarction and ischemic stroke. Aspirin and clopidogrel are most routinely used anti-platelet agents for the management of CVDs, but these agents irreversibly inhibit platelet activation with potentially devastating unwanted effects such as hemorrhages. In present study we reported that ADTM, a novel cardioprotective compound synthesized in our own laboratory^6^, displayed specific binding for several members of the PDI family. By using a biotin-conjugated ADTM analogue (BAA) as bait and chemical proteomic analysis of the bound protein complexes, we identified eight potential interacting protein targets of ADTM (Supplementary Table S1). With further validation using immunoblot analysis the identity of major targets were confirmed as members of the PDI family, including ERp72, ERp57 and ERp5. In particular, ADTM showed highest potency in inhibiting the redox activity of ERp57 in enzymatic assay *in vitro*. Furthermore, untagged-ADTM could compete with BAA for the binding of ERp57, providing evidence that ADTM is a competitive ligand at ERp57. We further demonstrated that ADTM displayed concentration-dependent inhibition in ADP-induced platelet aggregation and AA-induced platelet aggregation *in vitro*. This result is in line with the observation that ERp57 has been recently implicated to play crucial roles in mediating platelet aggregation, homeostasis and thrombosis^18^,^19^,^20^. It has also been reported that ERp57 present on platelet surface were activated by staphylococcal extracellular adhesive protein resulting in arteriosclerosis in *Staphylococcus aureus* infection, and the inhibition of ERp57 could suppressed platelet aggregation^30^.

**Figure 9:**
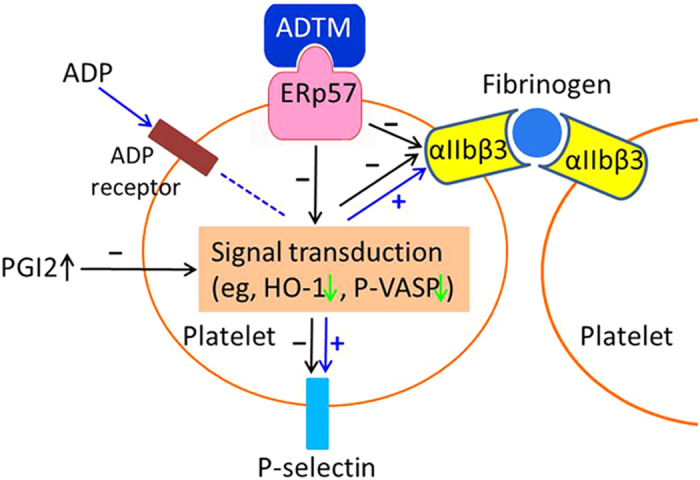
Proposed mechanism of ADTM anti-platelet aggregation. In part, ADTM binds with ERp57 and inhibits its redox activity, inhibits the activation of αIIbβ3 and the expression of P-selection and enhances the expression of HO-1 and phosphorylation of VASP. The crosstalk between ERp57 and αIIbβ3 is involved in the biological action of ADTM. In addition, ADTM reverses the decreased level of 6-Keto-PGF1α, a stable metabolite of PGI2 in the FeCl_3_-induced thrombosis model *in vivo*.

We further demonstrated that ADTM concentration-dependently inhibited the ADP-induced expression of P-selectin and activation of αIIbβ3 integrin *in vitro*. P-selectin is an adhesion molecule expressed on the surface of platelet and is an important marker for platelet activation. αIIbβ3 integrin is the most abundant adhesion receptor on the surface of circulating platelets and has been reported to mediate platelet aggregation through strong interactions with adhesive proteins such as fibrinogen, von Willebrand factor and fibronectin^31^. We further demonstrated that ERp57 bound to αIIbβ3 directly by using co-immunoprecipitation, and ADTM disrupted ERp57/αIIbβ3 interaction, as shown by the dissociation of the complex (Fig. 5c). In line with this, ERp57 has been reported to directly interact with αIIbβ3 on platelet surface and regulate its function in platelet aggregation^18^,^19^,^20^. Recent study has shown that disulfide bond exchanges in αIIbβ3 is required for activation and post-ligation signaling during clot retraction^32^, and the modulation of functional disulfide bonds is being explored for blood protein control^33^. Our results suggested that ADTM produced its anti-thrombotic action via the modulation of disulfide bond by interacting with ERp57 (Fig. 9).

The inhibitory action of ADTM on platelet integrin αIIbβ3 activation was furthered evidenced by the increase of the phosphorylation of VASP after ADTM pretreatment. The phosphorylation VASP is involved in the down-regulation of platelet aggregation accompanied by inhibition of platelet integrin αIIbβ3 activation^27^,^28^. We also observed that ADTM increased the expression of HO-1 in platelets. This is coherent with our previous finding that HO-1 activation has a key role in ADTM cardioprotective effect in H9c2 cardiomyoblast cells *in vitro* and in the myocardium *in vivo*^7^. HO-1 is a potential therapeutic target in treating thrombotic disease and HO-1 knockout mice demonstrated acute thrombus formation in response to hypoxia^34^. Previous studies reported that dengue virus glycoprotein-1 (DV-NS1) antibody inhibited PDI activity and blocked platelet aggregation accompanied by an increase in HO-1^35^,^36^. Furthermore, redox reactions have been shown to play crucial roles in platelet aggregation in a glutathione peroxidase-3 (GPx-3)-deficient mouse model. GPx-3 is an important anti-oxidant enzyme and redox sensor in plasma and GPx-3-deficiency impaired normal platelet inhibition and promoted thrombosis due to enhanced reactive oxygen species flux and decreased nitric oxide bioavailability^37^. In our *in vivo* studies, we observed that ADP-induced platelet aggregation was significantly compromised (>40% reduction) in rats treated with ADTM (20 mg/kg). The anti-thrombotic effect of ADTM was further evidenced by the reduction of platelet activity and thrombus formation in the ferric chloride (FeCl_3_)-induced venous thrombosis assay in the rat. The major metabolites of ADTM *in vivo* were 2-hydroxymethy-3, 5, 6-trimethylpyrazin (TMP-OH) and DSS^38^. We have checked the *in vivo* activity of DSS and TMP. Interestingly, the results showed that ADTM exhibited stronger antiplatelet and anti-thrombotic activities when compared to DSS and TMP, alone or in combination in ADP-induced platelet aggregation and FeCl_3_-ionduced thrombosis model, respectively (Figs. 7 and 8). The precise mechanisms of this action conferred by ADTM would be another interesting question that is worth investigating in the future. Recently there are accumulating reports that the inhibition of protein disulfide isomerases including PDI and ERp57 could block thrombus formation in various *in vivo* models and suggest that this protein family represents an important novel class of anti-thrombotic target^39^,^40^,^41^,^42^,^43^. Preclinical studies demonstrate that deficiency in platelet ERp57 resulted in the increased tail bleeding times and delayed thrombus formation^20^, while PDI is unable to compensate for the absence of platelet ERp57^20^ and other study also showed that the blockade of ERp57 with specific antibody further inhibited platelet aggregation in PDI deficient platelets^16^. These independent studies further provided evidences that ERp57 and PDI have distinct roles in the mediation of platelet function.

Taken together, our present study reported the anti-thrombotic action of ADTM with both *in vitro* and *in vivo* data. ADTM exhibited comparable anti-thrombotic properties as aspirin and clopidogrel. The results suggested that the anti-thrombotic action of ADTM is mediated through disrupting the interaction between ERp57 and αIIbβ3 possibly by blocking the action of ERp57 on disulfide bonding (Fig. 9). Our data provided a rationale for the further development of ADTM as anti-thrombotic agent targeting the underlying mechanism involving ERp57 and αIIbβ3. Furthermore, as a competitive ligand at ERp57, ADTM presents as a promising compound for the development of versatile anti-thrombotic agents. Our data also provide insights in novel strategies for the development of drugs targeted ERp57 for anti-thrombotic.

## Methods

### Materials

ADTM and BAA were synthesized at Jinan University, China. DSS and TMP were of analytical pure grade, and obtained from Xi’an Honson Biotechnology (China) and Shanghai Banghai Chemical Company (China), respectively. Human recombinant ERp57 was obtained from Abcam (Cambridge, UK), PDI and ERp72 were purchased from Enzo Life Sciences (Exeter, UK). Insulin, DTT, AA and ADP were obtained from Sigma Aldrich (St. Louis, MO, USA).

### Preparation of platelet-rich plasma

All animal experiments were approved by the Animal Care and Experimentation Committee of Jinan University and were performed in accordance with the approved guidelines. Sprague-Dawley rats were anesthetized with 10% chloral hydrate, and blood was obtained by an arterial puncture. Whole blood was anticoagulated with citrate (3.8%; 1:9, v/v) and centrifuged at 200 g for 8 min at room temperature to obtain platelet-rich plasma (PRP). The residue was centrifuged at 550 g for 5 min to obtain platelet-poor plasma (PPP).

### Protein preparation from platelets

Washed platelets were lysed using a dounce homogenizer in NP-40 lysis buffer (Beyotine, China) with 1 mM phenylmethylsulfonyl fluoride (PMSF). Platelet lysates were centrifuged at 12,500 g for 20 min at 4 °C, and the supernatant was collected and stored at −80 °C until further analysis.

### NeutrAvidin Agarose Resin pull-down with BAA

Platelet lysates (3 μg/μl) were exposed to NeutrAvidin Agarose Resin (Pierce Biotech., Rockford, IL, USA) for 2 h at 4 °C and then centrifuged for 3 min at 2,500 g. The resin was discarded to remove endogenous biotin. Aliquots of supernatants (300 μl) were first treated with DMSO or ADTM (600 μM) for 2 h, and then incubated with BAA (600 μM) for another 2 h. The supernatant was combined with NeutrAvidin Agarose Resin and allowed to shake overnight at 4 °C as described previously^44^. To eliminate the nonspecific combination with compound or agarose resin before protein analysis, 0.1% DMSO was added to one aliquot of lysate as a negative control.

### Nano LC-MS/MS analysis

The compound-protein complex bound to agarose resin was digested with trypsin. The digested peptides were analyzed with 1D nano-RP LC-MS/MS. Acquired MS data were analyzed using the Paragon algorithm in ProteinPilot 4.0 software (Applied Biosystems, Framingham, MA, USA). The identified peptides from the Paragon algorithm were grouped into minimal non-redundant protein sets by the ProGroup algorithm of the software (details of this method can be found in Supplementary Methods).

### Immunoblot analysis

Proteins eluted from the resin were subjected to SDS-PAGE and then transferred to a PVDF membrane for immunoblot (details in Supplementary Methods).

### Insulin reduction assay

PDI activity was measured using an assay that measured the catalytic reduction of insulin, as described previously with minor modifications^39^,^45^. Briefly, insulin (1 mg/ml) was freshly prepared in assay buffer (100 mM potassium phosphate, 1 mM EDTA (pH 7.4) and 10 μM dithiothreitol). 50 μl of insulin was incubated in the presence of 10 μl of enzyme and varying concentrations of *compound*, and then 10 μl of 0.1 mM dithiothreitol was added. The increase in turbidity was monitored at 620 nm using a microplate reader (Perkin-Elmer, Singapore) at 25 °C for 60 min. PDI, ERp57, and ERp72 were all used at 0.04 mg/ml. PDI family enzyme inhibition in the presence of compound was determined by the following formula: enzyme inhibition (%) = [1–(OD_[compound + PDI + DTT]_–OD_[DTT]_)/(OD_[PDI + DTT]_–OD_[DTT]_)] × 100%.

### Platelet aggregation *in vitro*

The inhibitory effect of ADTM was examined as previously described with modifications^39^. Briefly, platelets were incubated with the indicated concentrations of compounds or 0.1% DMSO at 37 °C for 5 min with shaking. Aggregation was induced by ADP (10 μM) or AA (200 μM) and measured using a platelet aggregometer (SC-2000, Saikexide Instrument Co. Ltd. China). Inhibition of aggregation was calculated using the following general formula: inhibition of aggregation = (rate of aggregation in vehicle group – rate of aggregation in treated group)/rate of platelet aggregation in vehicle group × 100%.

### Determination of human platelet activation by flow cytometry

Flow cytometry and immunoprecipitation studies involving human platelets were approved by Jinan University with informed consent in accordance with the World Medical Association Declaration of Helsinki and carried out in accordance with the approved guidelines. Flow cytometry was performed as described in the Supplementary Methods. PE conjugated anti-CD62P (P-selectin) antibody and FITC conjugated anti-PAC-1 (activated αIIbβ3) antibody were used to detect platelet activation markers after exposure to ADP in the presence of DMSO (0.1%) or different concentrations of ADTM. An isotype-matched IgG1 PE/FITC was used as a negative control.

### Immunoprecipitation

To determine ERp57-bound αIIbβ3 contents, whole platelet lysates were incubated with anti-αIIbβ3 antibody overnight at 4 °C under agitation. The antigen-antibody complex was pulled down after incubation for 4 h at 4 °C with protein G-agarose (Cell Signaling Technology) as described previously^46^. Immune complexes were boiled for 5 min with 2 × loading buffer followed by immunoblot analysis with antibodies for ERp57 and αIIbβ3.

### Rat platelet aggregation-induced by ADP *in vivo*

To study the effects of ADTM on platelet aggregation *in vivo*, ADTM (5–20 mg/kg) in comparison with DSS (10 mg/kg), TMP (6 mg/kg), DSS (10 mg/kg) + TMP (6 mg/kg) and clopidogrel (18 mg/kg) were administered daily by IV injection for 5 days, respectively (detailed methodology in Supplementary Methods). Platelet aggregation was monitored for 5 min and the maximum aggregation inhibition (%) was calculated.

### FeCl_3_-induced inferior vena cava thrombosis in rat

Rats weighing 180 g to 220 g were anesthetized by intraperitoneal administration of 10% chloral hydrate. One hour post-treatment, the medioventral line in the rats was cut and the inferior vena cava was surgically exposed by blunt dissection. 1 × 1 cm filter paper, soaked in 50% FeCl_3_ solution, was placed under and above the exposed inferior vena cava for 15 min. Subsequently, the thrombosed inferior vena cava was carefully dissected, and the weight (mg) of wet thrombus harvested from the inferior vena cava was measured as described previously^47^.

### Evaluation of plasma 6-Keto-PGF_1α_ levels in rat

The concentration of 6-keto-PGF_1α_ present in the supernatant was measured using a radioimmunoassay kit (New England Nuclear Dupont, Boston, MA) according to the manufacturer’s instructions. Blood was collected into plastic tubes containing 10% EDTA-Na_2_. Plasma was prepared by centrifuging blood at 550 g for 10 min. Plasma concentrations of 6-keto-PGF_1α_ were determined by radioimmunoassay and expressed as pg/ml.

### Statistical analysis

All assays were performed at least three times. Data are presented as mean ± SD. The groups were compared using one-way ANOVA followed by Tukey’s multiple comparison tests using the statistics module of Graph Pad Prism 5.0. A value of *P* < 0.05 was considered statistically significant.

## Additional Information

**How to cite this article**: Cui, G. *et al*. Novel anti-thrombotic agent for modulation of protein disulfide isomerase family member ERp57 for prophylactic therapy. *Sci. Rep*. **5**, 10353; doi: 10.1038/srep10353 (2015).

## Supplementary Material

Supplementary Information

## References

[b1] Roger V. L. . Heart disease and stroke statistics—2012 update: a report from the American Heart Association. Circulation 125, e2–e220 (2012). 22179539 10.1161/CIR.0b013e31823ac046 PMC4440543

[b2] Martinelli I., Bucciarelli P., & Mannucci P. M. Thrombotic risk factors: basic pathophysiology. Crit Care Med 38, S3–9 (2010). 20083911 10.1097/CCM.0b013e3181c9cbd9

[b3] Hansson G. K., & Klareskog L. Pulling down the plug on atherosclerosis: cooling down the inflammasome. Nat Med 17, 790–791 (2011). 21738158 10.1038/nm0711-790

[b4] Investigators AWGotA . Clopidogrel plus aspirin versus oral anticoagulation for atrial fibrillation in the Atrial fibrillation Clopidogrel Trial with Irbesartan for prevention of Vascular Events (ACTIVE W): a randomised controlled trial. Lancet 367, 1903–1912 (2006). 16765759 10.1016/S0140-6736(06)68845-4

[b5] Investigators A . Effect of clopidogrel added to aspirin in patients with atrial fibrillation. N Engl J Med 360, 2066–2078 (2009). 19336502 10.1056/NEJMoa0901301

[b6] Cui Q. . Design, synthesis, and preliminary cardioprotective effect evaluation of danshensu derivatives. Chem Biol Drug Des 84, 282–291 (2014). 24581174 10.1111/cbdd.12312

[b7] Cui G. . A novel Danshensu derivative confers cardioprotection via PI3K/Akt and Nrf2 pathways. Int J Cardiol 168, 1349–1359 (2013). 23290949 10.1016/j.ijcard.2012.12.012

[b8] Tian P. Convergence: Where West meets East. Nature 480, S84–86 (2011). 22190086 10.1038/480S84a

[b9] Jia Y., Huang F., Zhang S., & Leung S. W. Is danshen (Salvia miltiorrhiza) dripping pill more effective than isosorbide dinitrate in treating angina pectoris? A systematic review of randomized controlled trials. Int J Cardiol 157, 330–340 (2012). 21251721 10.1016/j.ijcard.2010.12.073

[b10] Cheng T. O. Cardiovascular effects of Danshen. Int J Cardiol 121, 9–22 (2007). 17363091 10.1016/j.ijcard.2007.01.004

[b11] Zhou L. M., Zuo Z., & Chow M. S. S. Danshen: An overview of its chemistry, pharmacology, pharmacokinetics, and clinical use. J Clin Pharmacol 45, 1345–1359 (2005). 16291709 10.1177/0091270005282630

[b12] Essex D. W. Redox control of platelet function. Antioxid Redox Signal 11, 1191–1225 (2009). 19061441 10.1089/ars.2008.2322

[b13] Furie B., Flaumenhaft R. Thiol isomerases in thrombus formation. Circ Res 114, 1162–1173 (2014). 24677236 10.1161/CIRCRESAHA.114.301808 PMC4067134

[b14] Cho J. Protein disulfide isomerase in thrombosis and vascular inflammation. J Thromb Haemost 11, 2084–2091 (2013). 24118938 10.1111/jth.12413 PMC4076787

[b15] Cho J., Furie B. C., Coughlin S. R., & Furie B. A critical role for extracellular protein disulfide isomerase during thrombus formation in mice. J Clin Invest 118, 1123–1131 (2008). 18292814 10.1172/JCI34134 PMC2248441

[b16] Kim K. . Platelet protein disulfide isomerase is required for thrombus formation but not for hemostasis in mice. Blood 122, 1052–1061 (2013). 23788140 10.1182/blood-2013-03-492504 PMC3739031

[b17] Reinhardt C. . Protein disulfide isomerase acts as an injury response signal that enhances fibrin generation via tissue factor activation. J Clin Invest 118, 1110–1122 (2008). 18274674 10.1172/JCI32376 PMC2242616

[b18] Holbrook L. M. . The platelet-surface thiol isomerase enzyme ERp57 modulates platelet function. J Thromb Haemost 10, 278–288 (2012). 22168334 10.1111/j.1538-7836.2011.04593.x PMC3444690

[b19] Wu Y. . The disulfide isomerase ERp57 mediates platelet aggregation, hemostasis, and thrombosis. Blood 119, 1737–1746 (2012). 22207737 10.1182/blood-2011-06-360685 PMC3286349

[b20] Wang L. . Platelet-derived ERp57 mediates platelet incorporation into a growing thrombus by regulation of the alphaIIbbeta3 integrin. Blood 122, 3642–3650 (2013). 24030382 10.1182/blood-2013-06-506691 PMC3837513

[b21] Ferrari D. M., Soling HD. The protein disulphide-isomerase family: unravelling a string of folds. Biochem J 339 **(Pt 1)**, 1–10 (1999). 10085220 PMC1220120

[b22] Ravanat C. . GPV is a marker of *in vivo* platelet activation—study in a rat thrombosis model. Thromb Haemost 83, 327–333 (2000). 10739394

[b23] Moog S. . Platelet glycoprotein V binds to collagen and participates in platelet adhesion and aggregation. Blood 98, 1038–1046 (2001). 11493449 10.1182/blood.v98.4.1038

[b24] Freedman J. E. Oxidative stress and platelets. Arterioscler Thromb Vasc Biol 28, s11–16 (2008). 18174453 10.1161/ATVBAHA.107.159178

[b25] Corazzari M. . Targeting homeostatic mechanisms of endoplasmic reticulum stress to increase susceptibility of cancer cells to fenretinide-induced apoptosis: the role of stress proteins ERdj5 and ERp57. Br J Cancer 96, 1062–1071 (2007). 17353921 10.1038/sj.bjc.6603672 PMC2360126

[b26] Laurindo F. R., Pescatore L. A., Fernandes Dde C. Protein disulfide isomerase in redox cell signaling and homeostasis. Free Radic Biol Med 52, 1954–1969 (2012). 22401853 10.1016/j.freeradbiomed.2012.02.037

[b27] Massberg S. . Enhanced *in vivo* platelet adhesion in vasodilator-stimulated phosphoprotein (VASP)-deficient mice. Blood 103, 136–142 (2004). 12933589 10.1182/blood-2002-11-3417

[b28] Horstrup K. . Phosphorylation of Focal Adhesion Vasodilator-Stimulated Phosphoprotein at Ser157 in Intact Human Platelets Correlates with Fibrinogen Receptor Inhibition. Eur J Biochem 225, 21–27 (1994). 7925440 10.1111/j.1432-1033.1994.00021.x

[b29] Peng L. . Induction of heme oxygenase-1 expression inhibits platelet-dependent thrombosis. Antioxid Redox Signal 6, 729–735 (2004). 15242554 10.1089/1523086041361677

[b30] Bertling A. . Staphylococcal extracellular adherence protein induces platelet activation by stimulation of thiol isomerases. Arterioscler Thromb Vasc Biol 32, 1979–1990 (2012). 22539594 10.1161/ATVBAHA.112.246249

[b31] Hynes R. O. Integrins: bidirectional, allosteric signaling machines. Cell 110, 673–687 (2002). 12297042 10.1016/s0092-8674(02)00971-6

[b32] Mor-Cohen R., Rosenberg N., Averbukh Y., Seligsohn U., & Lahav J. Disulfide bond exchanges in integrins alphaIIbbeta3 and alphavbeta3 are required for activation and post-ligation signaling during clot retraction. Thromb Res 133, 826–836 (2014). 24560420 10.1016/j.thromres.2014.02.001

[b33] Butera D., Cook K. M., Chiu J., Wong J. W., & Hogg P. J. Control of blood proteins by functional disulfide bonds. Blood 123, 2000–2007 (2014). 24523239 10.1182/blood-2014-01-549816 PMC3968386

[b34] Wu M. L., Ho Y. C., & Yet S. F. A central role of heme oxygenase-1 in cardiovascular protection. Antioxid Redox Signal 15, 1835–1846 (2011). 21091076 10.1089/ars.2010.3726

[b35] Cheng H. J. . Anti-dengue virus nonstructural protein 1 antibodies recognize protein disulfide isomerase on platelets and inhibit platelet aggregation. Mol Immunol 47, 398–406 (2009). 19822367 10.1016/j.molimm.2009.08.033

[b36] Immenschuh S. . Antibodies against dengue virus nonstructural protein-1 induce heme oxygenase-1 via a redox-dependent pathway in human endothelial cells. Free Radic Biol Med 54, 85–92 (2013). 23103292 10.1016/j.freeradbiomed.2012.10.551

[b37] Jin R. C. . Glutathione peroxidase-3 deficiency promotes platelet-dependent thrombosis *in vivo*. Circulation 123, 1963–1973 (2011). 21518981 10.1161/CIRCULATIONAHA.110.000034 PMC3107543

[b38] Li S. . Pharmacokinetic and Metabolic Studies of ADTM: A Novel Danshensu Derivative Confers Cardioprotection by HPLC-UV and LC-MS/MS. J Chromatogr Sci, 10.1093 (2015). 10.1093/chromsci/bmu133 25609599

[b39] Jasuja R. . Protein disulfide isomerase inhibitors constitute a new class of antithrombotic agents. J Clin Invest 122, 2104–2113 (2012). 22565308 10.1172/JCI61228 PMC3366406

[b40] Kennedy D. R. . Development Of Second Generation Thiol Isomerase Inhibitors To Prevent Thrombus Formation. Blood 122, 926–926 (2013).

[b41] Flaumenhaft R. Protein disulfide isomerase as an antithrombotic target. Trends Cardiovasc Med 23, 264–268 (2013). 23541171 10.1016/j.tcm.2013.03.001 PMC3701031

[b42] Loving G. S., Caravan P. Activation and retention: a magnetic resonance probe for the detection of acute thrombosis. Angew Chem Int Ed Engl 53, 1140–1143 (2014). 24338877 10.1002/anie.201308607 PMC4041297

[b43] Zhou J. . The disulfide isomerase ERp57 is required for fibrin deposition *in vivo*. J Thromb Haemost 12, 1890–1897 (2014). 25156521 10.1111/jth.12709 PMC4229395

[b44] Liu C. X. . Adenanthin targets peroxiredoxin I and II to induce differentiation of leukemic cells. Nat Chem Biol 8, 486–493 (2012). 22484541 10.1038/nchembio.935

[b45] Antoniou A. N. . The oxidoreductase ERp57 efficiently reduces partially folded in preference to fully folded MHC class I molecules. EMBO J 21, 2655–2663 (2002). 12032078 10.1093/emboj/21.11.2655 PMC126025

[b46] Yang S. . Pellino3 targets RIP1 and regulates the pro-apoptotic effects of TNF-alpha. Nat Commun 4, 2583 (2013). 24113711 10.1038/ncomms3583

[b47] Hennan J. K. . Effect of tiplaxtinin (PAI-039), an orally bioavailable PAI-1 antagonist, in a rat model of thrombosis. J Thromb Haemost 6, 1558–1564 (2008). 18624980 10.1111/j.1538-7836.2008.03063.x

