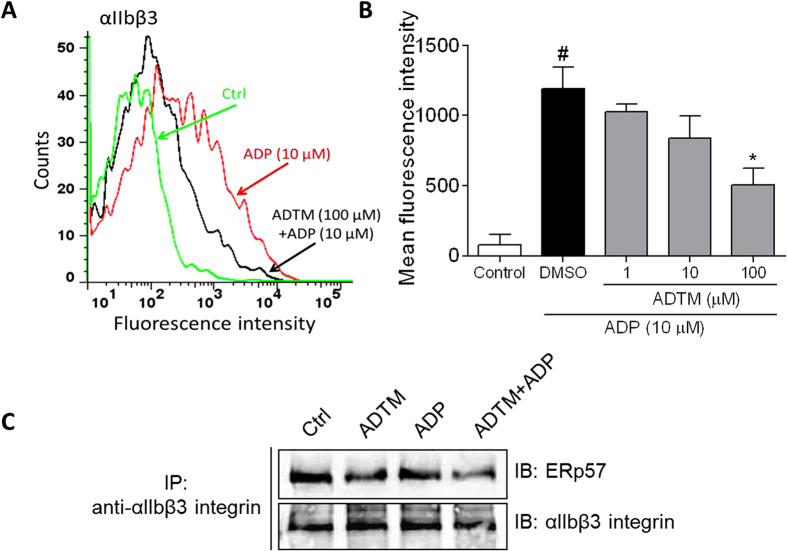# Corrigendum: Novel anti-thrombotic agent for modulation of protein disulfide isomerase family member ERp57 for prophylactic therapy

**DOI:** 10.1038/srep13509

**Published:** 2015-09-02

**Authors:** Guozhen Cui, Luchen Shan, Lin Guo, Ivan Keung Chu, Guohui Li, Quan Quan, Yun Zhao, Cheong Meng Chong, Zaijun Zhang, Pei Yu, Maggie Pui Man Hoi, Yewei Sun, Yuqiang Wang, Simon MingYuen Lee

Scientific Reports 5: Article number: 1035310.1038/srep10353; published online: 06032015; updated: 09022015.

In this Article, Fig. 5 is a duplication of Fig. 7. The correct Fig. 5 appears below as Fig. 1.

## Figures and Tables

**Figure 1:**