# Supplementary material for: Novel anti-thrombotic agent for modulation of protein disulfide isomerase family member ERp57 for prophylactic therapy
Source: Sci Rep. 2015 Jun 3;5:10353. doi: 10.1038/srep10353 (PMC4650696; doi:10.1038/srep10353)
Supplement: Supplementary Information [file srep10353-s1.doc]

**Novel anti-thrombotic agent for modulation of protein disulfide isomerase family member ERp57 for prophylactic therapy**

Guozhen Cui^1,5*^, Luchen Shan^2,4*^, Lin Guo^2^, Ivan Keung Chu^3^, Guohui Li^3^, Quan Quan^3^, Yun Zhao^3^, CheongMeng Chong^1^, Zaijun Zhang^2^, Pei Yu^2^, Maggie Pui Man Hoi^1^, Yewei Sun^2^, Yuqiang Wang^2^ & Simon MingYuen Lee^1^

^1^State Key Laboratory of Quality Research in Chinese Medicine and Institute of Chinese Medical Sciences, University of Macau, Macao, China, ^2^Institute of New Drug Research, Collage of Pharmacy, Jinan University, Guangzhou, China, ^3^Department of Chemistry, The University of Hong Kong, Hong Kong, China, ^4^Key Laboratory of Cardiovascular Medicine Research, Ministry of Education, Harbin Medical University, Harbin, China, ^5^Department of Bioengineering, Zhuhai Campus of Zunyi Medical University, Guangdong, Zhuhai, China.

^*^These authors contributed equally to this work.

Correspondence and requests for materials should be addressed to P.H. (email: maghoi.um@gmail.com) or to Y.S. (email: syw1984@163.com) or to M.L. (email: simonlee@umac.mo).

**Supplementary Table and Figures**

**Supplementary Table S1**

Platelet aggregation-associated proteins identified by pull down and mass spectrometry

| # | Accession no. | Protein name | Protein function |
| --- | --- | --- | --- |
| 1  2  3  4  5  6  7 | Q63081  P11598  G3V6T7  P04785  O08770  P07895  P22985 | ERp5  ERp57  ERp72  PDI  Glycoprotein 5  Superoxide dismutase  Xanthine oxidase | Plays a role in platelet aggregation and cell redox homeostasis.  Mediates platelet aggregation, thrombus formation and cell redox homeostasis  Mediates platelet aggregation and cell redox homeostasis  Mediates platelet aggregation and cell redox homeostasis  Mediates vWF-dependent platelet adhesion to blood vessels  Destroys superoxide anion radicals which are toxic to biological systems  Contributes to the generation of reactive oxygen species. |


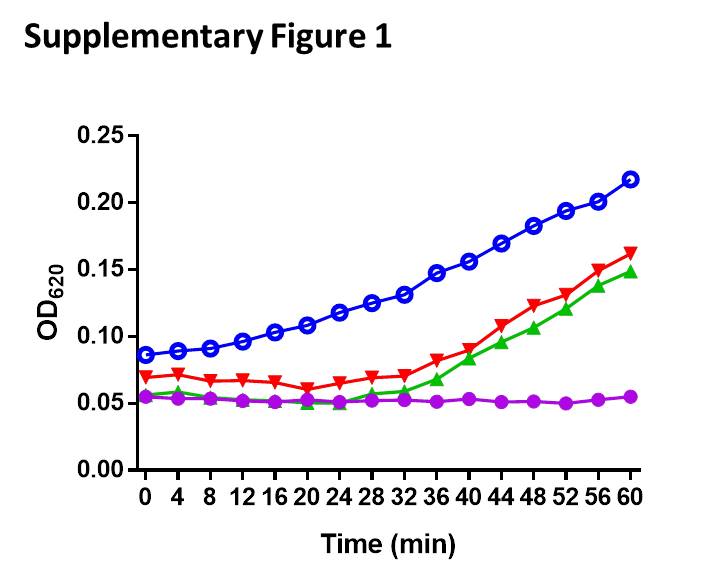


**Supplementary Figure 1 Irreversibility of inhibition of ERp57 by ADTM.** ERp57 (2 mg/ml) was incubated with 100 µM ADTM for 30 min and subsequently diluted 50-fold into assay buffer (green). Insulin reductase activity was similar to samples containing 0.04 mg/ml ERp57 in the presence of 100 µM ADTM. Green line indicates recovery of reductase activity after dilution of the ERp57-ADTM complex.


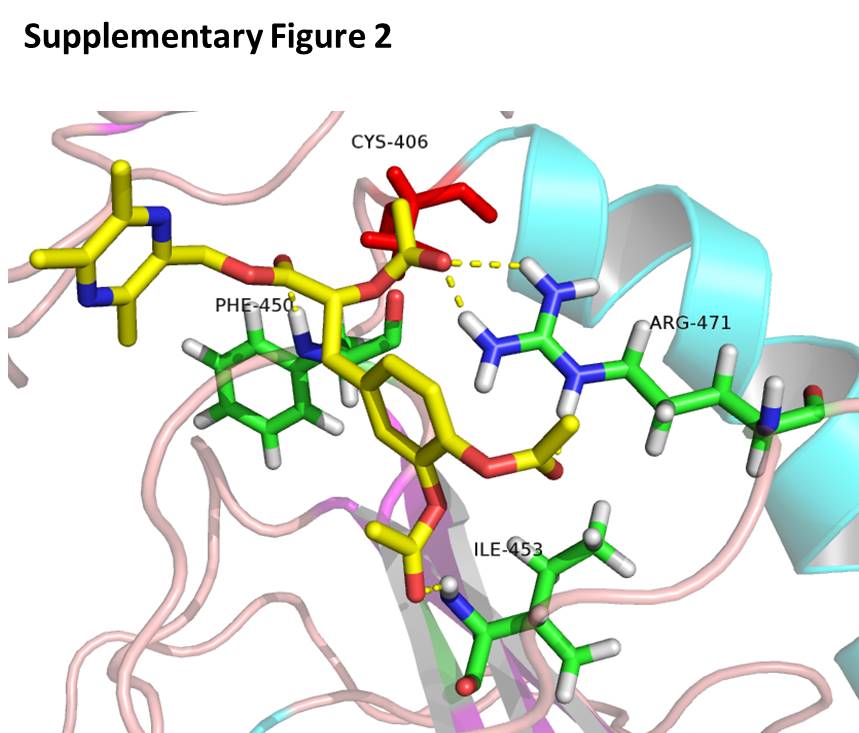


**Supplementary Figure 2 Molecular docking simulation of the interaction between ERp57 and ADTM.** The interaction between ERp57 and ADTM was viewed by the representative structure (best energy) of the energetically best structure clusters found by Autodock. The free binding energy to the active cavity of ERp57 (PDB: 3F8U) is -6.5 kcal/mol. ADTM may form hydrogen bonds with side chains of ARG47, ILE453 and PHE450, respectively, in the catalytic center (CYS406/CYS409) of ERp57. The yellow stick represents ADTM and the crystal structure of ERp57 domain with each monomer presented in ribbon diagram.


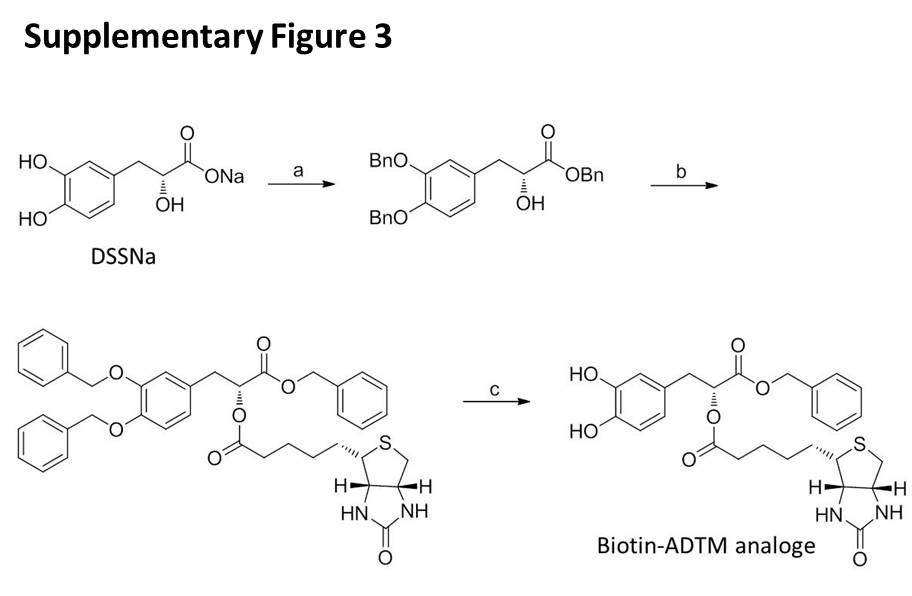


**Supplementary Figure 3 BAA compound synthesis.** The phenolic hydroxyl and carboxyl groups of DSS were first protected by benzyl group, and then biotin was conjugated to α-hydroxyl group using EDCI/HOBt/DMAP as catalyst, after removal of benzyl group of phenolic hydroxyl position to produce compound BAA.

**Supplementary Methods**

**Reversibility assay.** The recovery of reductase activity of ERp57 was evaluated as previously described method[^1^](#_ENREF_1). Briefly, ERp57 (2 mg/ml) was incubated with 100 µM ADTM for 30 min and subsequently diluted 50-fold into assay buffer (green). Insulin reductase activity was similar to samples containing 0.04 mg/ml ERp57 in the presence of 100 µM ADTM. Green line indicates recovery of reductase activity after dilution of the ERp57-ADTM complex.

**Sample preparation for chemical proteomics analysis.** Rat platelets were lysed using a Dounce homogenizer in NP-40 lysis buffer (Beyotine, China) with 1 mM phenylmethylsulfonyl fluoride (PMSF). Platelet lysates were centrifuged at 12,500 g for 20 min at 4°C, and the supernatant was collected and stored at -80°C until further usage. The platelet lysates (3 μg/μl) were exposed to beads (Pierce Biotech., Rockford, IL, USA) for 2 h at 4°C and then centrifuged for 3 min at 2,500 g. The resin was discarded to remove endogenous biotin. Aliquots of supernatants (300 μl) were treated with BAA (600 μM) for 2 h. The supernatant was combined with Beads and allowed to shake overnight at 4°C. To eliminate the nonspecific combination with compound or beads before protein analysis, 0.1% DMSO and Biotin were added to one aliquot of lysate as negative controls sample 1 and sample 2. The compound-protein bound to agarose resin were digested with trypsin in 37°C overnight and each injected to LC-MS, Sample 1 (DMSO) and sample 2 (Biotin) being the negative control and sample 3 being rich protein group.

**LC-MS and data analysis.** Nano-flow LC separation experiments were performed using Agilent 1200 series nano pump. Solvent A (2% ACN, 0.5% formic acid in water) and B (98% ACN, 0.5% formic acid in water) were used to provide pH 2 organic gradient. Reserve phase C_18_ separation with 60 min 5-35% solvent B gradient was applied for all three samples. TripleTOF 5600 system (AB SCIEX, Concord, ON, Canada) fitted with a Nanospray III source (AB SCIEX, Concord, ON, Canada); the parameters used were as follows: ion spray voltage, 2.8 kV; curtain gas, 30 psi; nebulizer gas, 6 psi; interface heater temperature, 150°C. For IDA, full scans were acquired within 250 ms over the range *m*/*z* 400-1250, followed by MS/MS scans of the 20 most abundant peaks that exceeded 125 counts per second and carried a charge between +2 to +5 in the range *m*/*z* 100-1500. The dynamic exclusion time of the acquired ions was set at 20 s. The acquired MS/MS data were analyzed using the Paragon algorithm in ProteinPilot 4.0 software (Applied Biosystems, Framingham, MA, USA)[^2^](#_ENREF_2). They were searched against theoretical spectra generated from sequences in Uniprot (www.uniprot.org). Uniprot Rat complete proteome database released in August 2011 (34,098 entries) was used.

­­­­­­­

**Platelet aggregation *in vitro.*** The inhibitory effect of ADTM was examined as previously described procedure with modification[^1^](#_ENREF_1). Briefly, PRP was suspended in Tyrode buffer (137 mM NaCl, 12 mM NaHCO_3_, 5.5 mM glucose, 2 mM KCl, 1 mM MgCl_2_, 0.3 mM NaHPO_4_, [pH 7.4]) and centrifuged at 200 g for 8 min at room temperature. The washed platelets (1×10^8^ platelets/ml in Tyrode buffer) were incubated with indicated concentrations of compounds (treated group) or 0.1% DMSO (vehicle group) at 37°C for 5 min in the presence of shaking. Aggregation was induced by ADP (10 μM) or AA (200 μM) and measured using a platelet aggregometer (SC-2000, Saikexide Instrument Co. Ltd. China). The inhibition of aggregation was calculated as the following general formular: inhibition of aggregation = (Rate of aggregation in vehicle group - Rate of aggregation in treated group)/Rate of platelet aggregation in vehicle group × 100%.

**Determination of platelet activation by flow cytometry.** Flow cytometry was performed as previous described method[^3^](#_ENREF_3). The anti-CD62P (P-selectin) antibody and anti-PAC-1 (activated GPIIb/IIIa) antibody were used to detect determine the platelet activation. Whole blood (10 μl) was incubated with Tyrode-HEPES buffer (70 μl) and pre-incubated for 10 min in the presence or absence of various concentrations of ADTM. The suspensions were then incubated for another 5 min at room temperature after the addition of ADP (10 μM). 25 μl of test sample from above was added to tubes containing 10 μl of each antibody (PAC-1-FITC, anti-CD62P-PE) or respective isotype control and incubated for 30 min at 37°C [in](app:ds:in) the [dark](app:ds:dark). Subsequently, Red Blood Cell Lysis Buffer was added to the sample and analyzed by flow cytometry (Becton-Dickinson, FACsort Systems, Bedford, MA, USA) with CELLQuest3.1 software. Histograms were produced by quantification of the fluorescence intensity of 10,000 platelets per experimental group, with peak height indicating the number of platelets with a given intensity.

**Rat platelet aggregation-induced by ADP *in vivo.*** Forty SD rats were divided randomly into eight groups for platelet aggregation study *in vivo*: vehicle group, ADTM groups (5, 10 and 20 mg/kg), clopidogrel group (18 mg/kg), DSS group (10 mg/kg), TMP (6 mg/kg), DSS+TMP (10 mg/kg DSS+6 mg/kg TMP), clopidogrel (18 mg/kg). Each group contained 6-10 animals. Compounds were administered by intravenous injection once daily. Blood was collected from abdominal aorta after 5 days. Platelet-rich plasma (PRP) was obtained by centrifuging blood at 200 g for 10 min at room temperature. The remaining blood was centrifuged at 550 g for another 10 min to obtain platelet-poor plasma (PPP). 290 μl of PRP and 100 μl solvent or compound solution was placed in a cuvette and stirred with rotor at 37°C for 5 min, and then 10 μl ADP (10 μM) was added. Aggregation tests were done in a platelet aggregometer (SC-2000, Saikexide Instrument Co. Ltd. China). PPP served as an appropriate blank. The maximum platelet aggregation rate within 5 min was measured. The percentage inhibition of platelet aggregation was calculated as follows: percentage inhibition (%) = (Rate of aggregation in vehicle group-Rate of aggregation in compound treated group)/Rate of platelet aggregation in vehicle group×100%.

**Preparation of BAA.** DSS was dissolved in acetone in a 25 ml round-bottom flask. The solution was stirred at room temperature, and then benzyl bromide and anhydrous potassium carbonate were added. The reaction mixture was refluxed for 24 h and was allowed to cool to room temperature. Solvent was removed in vacuo, and the crude product was purified by column chromatography (EtOAc/petroleum ether, 1/3, v/v) to give a crystalline product. This white compound was then dissolved in DMF, and Biotin, EDCI, HOBt, DMAP were added in this solution and stirred for 15 h under room temperature. 10% NaHCO_3_ solution was added, and the product was extracted with dichloromethane. The solution was dried over Na_2_SO_4_, and solvent was removed. The crude product was purified by column chromatography (Dichloromethane /Ethanol, 20/1, v/v) to give a colorless oil. This product was dissolved in dichloromethane, and then ethanethiol, boron trifluoride diethyl etherate were added in this solution to stir 3 h under room temperature. Water was added to quench the reaction and the product was extracted with dichloromethane which was then dried over Na_2_SO_4_, and solvent was removed. The crude product was purified by column chromatography (Dichloromethane /Ethanol, 10/1, v/v) to give BAA as a yellow powder. ^1^H-NMR (CDCl_3_, 300 MHz) δ: 1.36 (m, 2 H, CH_2_), 1.57 (m, 4 H, CH_2_), 2.35 (m, 2 H, CH_2_), 2.80 (m, 1 H, CH), 2.95 (d, *J* = 18 Hz, 2 H, CH_2_), 3.05 (m, 2 H, CH_2_), 4.21 (m, 1 H, CH), 4.37 (m, 1 H, CH), 5.10 (s, 2 H, CH_2_), 5.13 (s, 4 H, CH_2_), 5.23 (m, 1 H, CH), 5.45 (s, 1 H, NH), 6.01 (s, 1 H, NH), 6.72 (m, 1 H, CH, arom), 6.85 (m, 2 H, CH, arom), 7.29 (m, 15 H, CH, arom). MS (ESI) [M+H]^+^ m/z 695.6.

**Database Search and Data Analysis.** The acquired MS/MS data were analyzed using the Paragon algorithm in ProteinPilot 4.0 software (Applied Biosystems, Framingham, MA, USA) as previously described methods^2^. They were searched against Rattus noevegicous complete proteome database released in August 2011 (34,098 entries) by Uniprot (http://www.uniprot.org). In the search, trypsin was set as the enzyme used and rapid search mode was adopted. Precursor and product ion accuracy was preset by choosing the instrument option as TripleTOF 5600 in the software. The identified peptides from the Paragon algorithm were grouped into minimal non-redundant protein sets by the ProGroup algorithm of the software. For protein identifications, a minimal unused ProtScore of 1.3 with at least one peptide (confidence ≥95%) was necessary. The false discovery rate (FDR) analysis was performed using the PSPEP add-on function of Protein-Pilot based on a decoy database of reverse sequences[^4^](#_ENREF_4).

**Immunoblot analysis.** For analysis of phosphor-VASP and HO-1 levels, lysates from rat platelets were immunoblotted according to a previously described method[^5^](#_ENREF_5). Antibodies against phospho-VASP^ser157^, VASP and GAPDH were bought from Cell Signaling Technology (Beverly, MA, USA), anti-HO-1 monoclonal antibody was purchased from Abcam (Cambridge, MA, USA).

For validation of chemical proteomics data, resin bound proteins were washed four time in 0.5 ml lysis buffer and remaining complexes were resuspended in 2×SDS sample loading buffer, followed by a 5 min boil. Anti-ERp72, Anti-ERp57 and Anti-ERp5 antibodies were bought from Santa Cruz Biotechnology (Santa Cruz, CA). After incubation with the above mentioned primary antibodies, the membrane was incubated for 1 h with secondary horseradish peroxidase-conjugated antibody (Cell Signaling Technology, Beverly, MA). Proteins were detected by an advanced enhanced ECL system (GE Healthcare, Little Chalfont, UK).

**Molecular docking study.** Molecular docking of ADTM to ERp57 was performed using the three dimensional (3-D) crystal structure of human ERp5 (PDB: 3F8U) obtained from the Protein Data Bank. The software Autodock 4.2 was used for all dockings in this study as previously described method[^6^](#_ENREF_6). In general, the docking parameters for AutoDock were kept to their default values. The grid box was 20 Å × 20 Å × 20 Å, encompassing the active site cavity (CYS406/CYS409) of human ERp57. The docking results were ranked by the binding free energy. The binding modes with lowest binding free energy and the most cluster members were chosen for the optimum docking conformation. The binding results were illustrated as three dimensional (3-D) by PyMOL Molecular Graphics System Version 1.3 (Schrödinger, LLC).

**Supplementary References**

1. Jasuja R*, et al.* Protein disulfide isomerase inhibitors constitute a new class of antithrombotic agents. *J Clin Invest* **122**, 2104-2113 (2012).

2. Shilov IV*, et al.* The paragon algorithm, a next generation search engine that uses sequence temperature values and feature probabilities to identify peptides from tandem mass spectra. *Molecular & Cellular Proteomics* **6**, 1638-1655 (2007).

3. Wu Y*, et al.* The disulfide isomerase ERp57 mediates platelet aggregation, hemostasis, and thrombosis. *Blood* **119**, 1737-1746 (2012).

4. Tang WH, Shilov IV, Seymour SL. Nonlinear fitting method for determining local false discovery rates from decoy database searches. *J Proteome Res* **7**, 3661-3667 (2008).

5. Cui G*, et al.* A novel Danshensu derivative confers cardioprotection via PI3K/Akt and Nrf2 pathways. *Int J Cardiol* **168**, 1349-1359 (2013).

6. Morris GM*, et al.* AutoDock4 and AutoDockTools4: Automated docking with selective receptor flexibility. *J Comput Chem* **30**, 2785-2791 (2009).
